# SuperTac - tactile data super-resolution via dimensionality reduction

**DOI:** 10.3389/frobt.2025.1552922

**Published:** 2025-06-26

**Authors:** Neel Patel, Rwik Rana, Deepesh Kumar, Nitish V. Thakor

**Affiliations:** ^1^ Department of Mechanical Engineering, Indian Institute of Technology Gandhinagar, Gandhinagar, Gujarat, India; ^2^ School of Biomedical Engineering, Indian Institute of Technology (BHU) Varanasi, Varanasi, Uttar Pradesh, India; ^3^ Department of Biomedical Engineering, Johns Hopkins University, Baltimore, MD, United States

**Keywords:** mechanoreceptors, robotic finger, tactile sensor, tactile super-resolution, texture, variational autoencoder

## Abstract

The advancement of tactile sensing in robotics and prosthetics is constrained by the trade-off between spatial and temporal resolution in artificial tactile sensors. To address this limitation, we propose SuperTac, a novel tactile super-resolution framework that enhances tactile perception beyond the sensor’s inherent resolution. Unlike existing approaches, SuperTac combines dimensionality reduction and advanced upsampling to deliver high-resolution tactile information without compromising the performance. Drawing inspiration from the spatiotemporal processing of mechanoreceptors in human tactile systems, SuperTac bridges the gap between sensor limitations and practical applications. In this study, an in-house-built active robotic finger system equipped with a 4 × 4 tactile sensor array was used to palpate textured surfaces. The system, comprising a tactile sensor array mounted on a spring-loaded robotic finger connected to a 3D printer nozzle for precise spatial control, generated spatiotemporal tactile maps. These maps were processed by SuperTac, which integrates a Variational Autoencoder for dimensionality reduction and Residual-In-Residual Blocks (RIRB) for high-quality upsampling. The framework produces super-resolved tactile images (16 × 16), achieving a fourfold improvement in spatial resolution while maintaining computational efficiency for real-time use. Experimental results demonstrate that texture classification accuracy improves by 17% when using super-resolved tactile data compared to raw sensor data. This significant enhancement in classification accuracy highlights the potential of SuperTac for applications in robotic manipulation, object recognition, and haptic exploration. By enabling robots to perceive and interpret high-resolution tactile data, SuperTac marks a step toward bridging the gap between human and robotic tactile capabilities, advancing robotic perception in real-world scenarios.

## 1 Introduction

In traditional robotics, vision has been the primary sensory modality. However, as robots are increasingly deployed in unstructured environments and tasked with complex object manipulation, the sense of touch becomes indispensable. Tactile sensing is a critical component of robotic or prosthetic perception, enabling machines to interact with their surroundings through touch. It plays a pivotal role in applications such as object recognition, material classification, robotic manipulation, and haptic exploration (Pyo et al., 2021; Meribout et al., 2024). A robust sense of touch allows robots to perform tasks that require precise interaction, such as handling fragile objects or exploring intricate surfaces. Despite significant advancements, artificial tactile sensing systems remain limited in achieving the high resolution and efficiency of human touch perception. A key challenge is the trade-off between spatial and temporal resolution in tactile sensors. Increasing spatial resolution often reduces temporal resolution due to hardware constraints such as sampling rates and communication bandwidths (Wang et al., 2023; Huang et al., 2025).

The human tactile system provides an exemplary model for addressing this trade-off. Mechanoreceptors in human skin process tactile information with high spatial and temporal resolution, and the brain integrates this data to enable rapid and precise tactile perception (Johansson and Flanagan, 2009). Remarkably, the human tactile system achieves hyperacuity, distinguishing tactile stimuli separated by as little as 0.3 mm (Abraira and Ginty, 2013), finer than the receptive field of any single mechanoreceptor (approximately 2 mm). This capability arises from the population-level encoding of tactile information, where the spatiotemporal patterns of mechanoreceptor activation across neighborhoods are processed synergistically. This biological inspiration underscores the need for artificial systems capable of similar spatiotemporal processing, bridging the gap between sensor limitations and practical applications.

Previous research aimed at enhancing tactile resolution has predominantly concentrated on developing specialized sensor architectures and advanced fabrication techniques (Wang et al., 2023). For example, Lu et al. (2024) designed a biomimetic soft tactile sensor inspired by the Pacinian corpuscle, optimizing the soft silicone layer for super-resolution. Li et al. (2022) utilized a high-throughput laser manufacturing method to achieve fine spatial resolution (0.7 mm) with minimal crosstalk. Yan Y. et al. (2021) developed a flexible, self-powered triboelectric sensing array via laser direct writing on laser-induced graphene, enabling high-resolution (8 dpi) real-time sensing. Similarly, Zhang et al. (2022) introduced a fast-photocurable solid-state conductive ionoelastomer (SCIE) that supports high-resolution 3D printing of robust, stretchable tactile sensors. While these approaches have demonstrated impressive performance, they are inherently tied to specific sensor designs and fabrication processes. While these fabrication-centric strategies have advanced tactile sensor performance, they are often resource-intensive, sensor-specific, and limited in scalability. As tactile sensing applications expand across diverse platforms, there is a growing need for algorithmic approaches to super-resolution that can enhance tactile resolution independent of the underlying sensor hardware.

More recently, a new wave of tactile sensing strategies has focused on integrating various tactile super-resolution algorithms with existing or custom-made tactile sensors to address the limitation of traditional tactile sensors sensing (Yu and Liu, 2025). Few studies mimicked the tactile sensing and encoding strategy used by human mechanoreceptors (neuromorphic tactile sensing) to achieve high-resolution tactile sensing and showed applications in texture classification, edge detection and slip detection (Kumar et al., 2020; Sankar et al., 2019; Parvizi-Fard et al., 2021). Other studies used various machine learning based approach for tactile super-resolution. For instance, a deep neural network-based reconstruction framework, EIT-NN, was proposed to enhance the performance of electrical impedance tomography (EIT)-based sensors, improving spatial resolution and sensitivity while maintaining simplicity in sensor design (Park et al., 2021). Similarly, the Local Message Passing Network (LoMP) enabled high-resolution calibration of piezoresistive sensor arrays using limited single-touch data, addressing calibration challenges in multi-touch scenarios (Kim et al., 2021). Another significant development involves the use of soft magnetic skin for tactile sensing, which decouples normal and shear forces and achieves super-resolution through deep learning algorithms (Yan Z. et al., 2021). Another approach by Wu et al. (2022) introduces TactileSRCNN and TactileSRGAN, which adapt image super-resolution techniques such as CNNs and GANs to upscale low-resolution tactile patterns from taxel-based sensors by a factor of 100, enabling multi-point contact detection from a single tap. Another method (Oller et al., 2023) focuses on modeling the dynamics of deformable tactile membranes by combining 3D geometric data and proprioceptive feedback to predict sensor deformation and improve manipulation control. Similarly, (Ouyang et al., 2024) presents a high-resolution piezoresistive sensor array integrated with machine learning algorithms, achieving fine spatial and temporal resolution and demonstrating 98.9% accuracy in shape recognition. These studies collectively highlight the growing role of machine learning in achieving tactile super-resolution and precise pattern recognition. These advancements have enabled precise tactile feedback for tasks such as adaptive grasping and teleoperation, emphasizing the potential of combining advanced sensor designs with computational models.

Despite these innovations, a generalized approach to achieving tactile super-resolution using standard low-resolution sensors remains a challenging task. Existing methods often require specialized hardware or extensive calibration, limiting their scalability. To address these limitations, we propose SuperTac, a real-time, hardware-efficient tactile super-resolution framework that operates using standard low-resolution tactile sensors. By combining dimensionality reduction using a Variational Autoencoder (VAE) and advanced upsampling using Residual-In-Residual Blocks (RIRB), our method delivers fourfold spatial resolution improvement while maintaining over 50 frames per second (FPS) throughput. This makes SuperTac a promising step toward a generalizable framework for real-time tactile super-resolution, offering high performance without relying on complex hardware or visual sensors.

To validate the SuperTac framework, we designed an experimental setup featuring an in-house-built active robotic finger system equipped with a 4 × 4 tactile sensor array. This system palpated textured surfaces to generate spatiotemporal tactile maps as input data for the SuperTac network. We experimented with super-resolution outputs of 8 × 8, 16 × 16 and 32 × 32. Among these, the 16 × 16 resolution offered the best trade-off between the image detail and reconstruction quality, leading to significantly improved tactile representation. The results demonstrate a fourfold improvement in spatial resolution, producing 16 × 16 super-resolved (SR) tactile images.

While this study uses a tactile sensor similar in design to the one employed by Kumar et al. (2020), our objective and methodological approach are fundamentally different. Kumar et al. focused on encoding spatiotemporal features from low-resolution tactile data for classification tasks using standard neural networks. In contrast, our work introduces *SuperTac*, a novel framework that integrates dimensionality reduction via a Variational Autoencoder with Residual-In-Residual Blocks to achieve real-time super-resolution of tactile data. This enables multifold increase in spatial resolution, leading to significantly improved texture classification performance and enabling broader applications in high-resolution tactile perception. Additionally, SuperTac is designed to be sensor-agnostic and operates in real time, offering scalability that was not addressed in Kumar et al.‘s work.

The major contributions of this work include.

1. Development of a novel tactile super-resolution framework that integrates dimensionality reduction and advanced upsampling.
2. Demonstration of a fourfold improvement in tactile sensor resolution with computational efficiency suitable for online deployment.
3. Validation of the framework through an active robotic finger system for tactile data collection.
4. Significant improvement in texture classification accuracy using super-resolved tactile data.

This paper is organized as follows: Section 2 describes the materials and methods used in this study. Section 3 details the SuperTac framework, including its architectural components and training methodology. Section 4 presents the results and Section 5 discusses the findings. Finally, Section 5 concludes the paper with insights and future directions.

## 2 Materials and methods

### 2.1 Tactile sensor array

**FIGURE 1:**
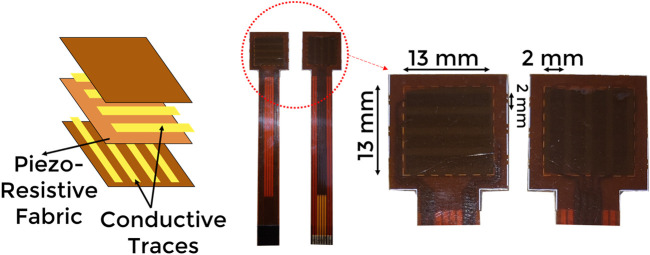
An exploded view of the tactile sensor array used in the experiment.

This study uses a fabric-based piezoresistive tactile sensor for the robotic palpation experiment. We use a similar tactile sensor previously used by Kumar et al. (2020). It is a 2D array of 16 tactile sensing elements (taxels) arranged in a 4 × 4 grid, within an area of 13 × 13 mm (Figure 1). A piezoresistive cloth is sandwiched between conductive traces arranged as rows and columns. The width of traces is 2 mm with a 1 mm spacing between consecutive traces, and therefore each taxel has a size of 2 mm × 2 mm. The tactile data were recorded at the sampling rate of 300 Hz per taxel.

### 2.2 Tactile stimuli and robotic palpation

#### 2.2.1 Design of tactile stimuli

**FIGURE 2:**
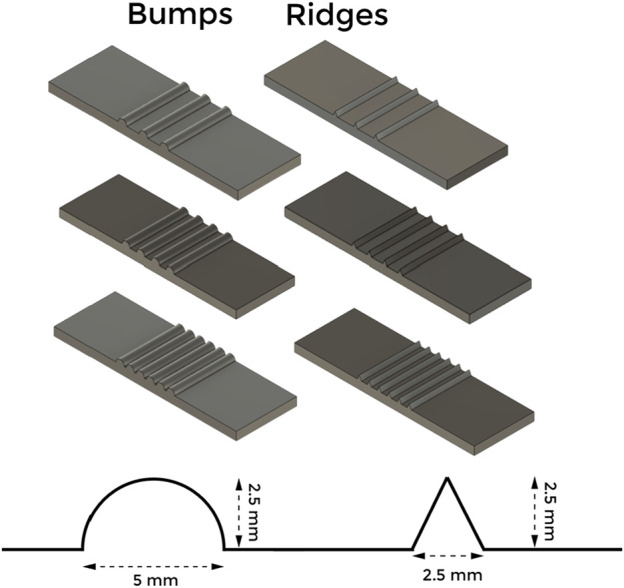
A schematic of the tactile stimulus. The height of each of the protrusion is 2.5 mm and the base width of the protrusion are 2.5 mm.

We used two categories of graded textures for our experiments, i.e., ridges and bumps. These textures were fabricated using a 3D printer and PLA plastic as the printing material. As shown in Figure 2, ridges are triangular protrusions, and bumps are semi-circular protrusions. The three textures of each type have a 12 mm, 6 mm, and 4 mm distance between each protrusion. Varying the distance between subsequent protrusions allows the dataset to be diverse, and the proposed network would learn to generate output as a convex combination of the known bumps and ridges.

#### 2.2.2 Design of robotic finger

**FIGURE 3:**
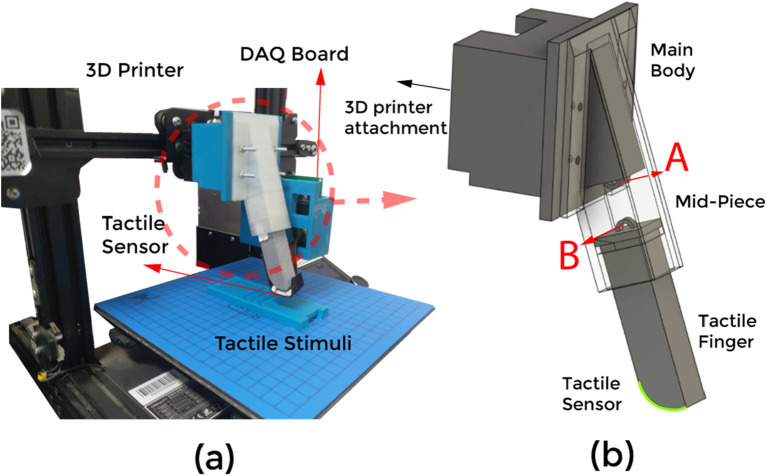
**(a)** The experimental setup with the finger attached to the 3D printer. **(b)** An enlarged view of the finger containing three parts; Main body, Mid-Piece and the finger. The suspension system is attached to points (A, B).

The tactile sensor is integrated into an in-house-built robotic finger system (Figure 3a). The finger consists of three components: the main body, mid-piece, and tactile fingertip (Figure 3b). A helical spring is incorporated into the design to function as a suspension system, enabling the finger to bend passively in response to external forces. This passive compliance allows the finger to conform smoothly to various terrains and surfaces. The stiffness of the spring ensures that the bending occurs in a controlled manner, providing stability during operation. To achieve precise spatial movement, the main body of the robotic finger is securely mounted onto the nozzle of a 3D printer.

### 2.3 Experimental protocol

**FIGURE 4:**
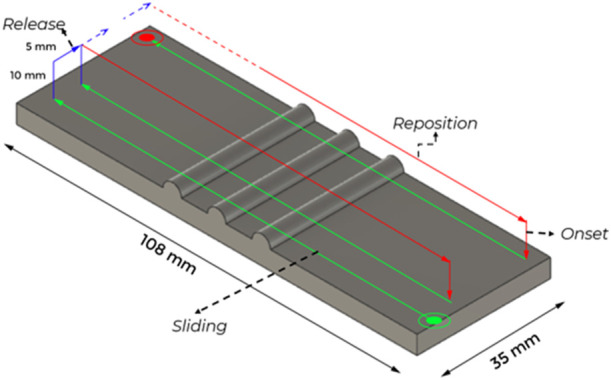
The tactile finger traverses on the tactile stimuli in 4 phases; Onset: Positioning the finger above the initial point. Sliding: Palpating the finger over the texture stimuli. Release: Breaking the contact of the finger and the stimuli and offsetting the finger for the next sliding action. Reposition: Bringing the finger to the next initial point which is at a horizontal offset to the previous initial point.

The entire experimental setup and data acquisition system has 4 phases. The phases are categorized according to the rectangular movement of the finger to palpate over textured plates. Figure 4 shows the four phases; onset, sliding, release, and re-position.

For each texture plate, eight sliding actions are performed to cover the width of the textured plate. Two trials were performed for each of eight sliding actions for each texture. The texture plates were fixed on the 3D printer base and the robotic finger palpated over the textured plates during the palpation. The tactile data were collected for sliding speeds at 5 mm/s and 10 mm/s.

### 2.4 Super-Tac algorithm

The proposed approach for tactile data super-resolution stems from the idea that points on an image depict their positions with respect to each other, and extracting these features helps capture information about the point and its surrounding. Along with the spatial component, the temporal features give us an idea about the context of the data in the current time step and use information from preceding time steps to extract underlying features. Combining both spatial and temporal feature extractors enable the extraction of rich features from the input sequence (Kumar et al., 2020). To capture the temporal features, a vast percentage of methods employ a sliding frames window along with neural network architectures like bidirectional recurrent convolutional networks (BRCN) (Jo et al., 2018) and long short-term memory networks (LSTM) (Huang et al., 2015). Our methodology combines the aspects of tactile sensing with image processing to generate super-resolution data. We have visualized tactile data in the form of images and used deep learning-based algorithms to achieve tactile super-resolution. We take inspiration from VAE to capture the latent features, which are simpler to process, and we modify the residual blocks presented in Zhang et al. (2018) as our upsampler. Thus, we intend to reduce the dimensions of the input data and then use the dimensionally reduced latent parameters to fabricate the SR image.

#### 2.4.1 Data visualization and pre-processing

The tactile sensor used in this study has 16 tactile sensing elements stacked in a grid manner, and therefore we visualize the data in 2 forms. The 16 channels are flattened along with the columns and rows, denoting the finger’s movement in the direction of columns and rows, respectively. “N” such flattened row-vectors are stacked over each other; N is the number of timesteps (Ts). Each row in the visualization corresponds to a particular timestep, and each cell in a row corresponds to tactile data from the 16 taxels.

The movement of the 3D printer nozzle is constrained to the horizontal plane only. The tactile sensor provided the output data in the range of 0 V–2.5 V, which was linearly mapped to a scale of [0–255], corresponding to an 8-bit resolution commonly used in image processing. We try to visualize the obtained tactile data in the form of 4 × 4 images, enabling us to apply the concepts from image processing in our analysis.

#### 2.4.2 Variational autoencoders

A Variational Autoencoder (VAE) (Kingma and Welling, 2014) provides a probabilistic manner for describing an observation in latent space. It has two main components; the encoder and the decoder. The latent parameters are sampled from the normal distribution using the mean and variance of the output of the encoder network. VAE is suited for dimensionality reduction of tactile sensors into latent features. Biological neurons pass the latent information in the form of neuronal spikes. Sorting the neuronal spikes based on a gaussian mixture model (GMM) (Souza et al., 2019) is well known and practiced. This suggests that the latent features can be represented by Gaussian curves or sampled from normal distributions. This is what VAE exactly does; proper training of VAE using Kullback–Leibler divergence Loss (KLD) loss ensures that latent features are sampled from the standard normal distribution (a particular case of a gaussian curve). Thus, VAE’s functionality of representing information is similar to the way actual biological neurons do and thus is a good fit for our network.

$$\left.\begin{array}{l} x=x1,x2,x3,x4\\ f\left(x_{1}\right)+h_{1}\left(x_{i}\right)=\mu_{i},f\left(x_{i}\right)+h_{2}\left(x_{i}\right)=\sigma_{i}\\ \;l_{r_{i}}\;∼Ɲ\left(\mu_{i},\sigma_{i}\right)=>l_{r_{i}}=\mu_{i}+\epsilon_{i}\sigma_{i}\\ LR=\left[l_{r_{1}},l_{r_{2}},l_{r_{3}},l_{r_{4}}\right]\\ Output=\left[g\left(l_{r_{1}}\right),g\left(l_{r_{2}}\right),g\left(l_{r_{3}}\right),g\left(l_{r_{4}}\right)\right] \end{array}\right\}$$ (1)

The Equation 1 is a simplified mathematical model for our context, *f* is the encoder network with input from the VAE stack $x_{i}\left|i\in \left\{1,2,3,4\right\}\right.$ , and h_1_ and h_2_ are identical but disjoint functions added to the final layer of the encoder function, whose weights and gradients are calculated separately to obtain the mean and variance. We sample from a normal distribution to obtain the latent representation *lr*_i_. Finally, *g* is the decoder network that takes the latent data as input.

**FIGURE 5:**
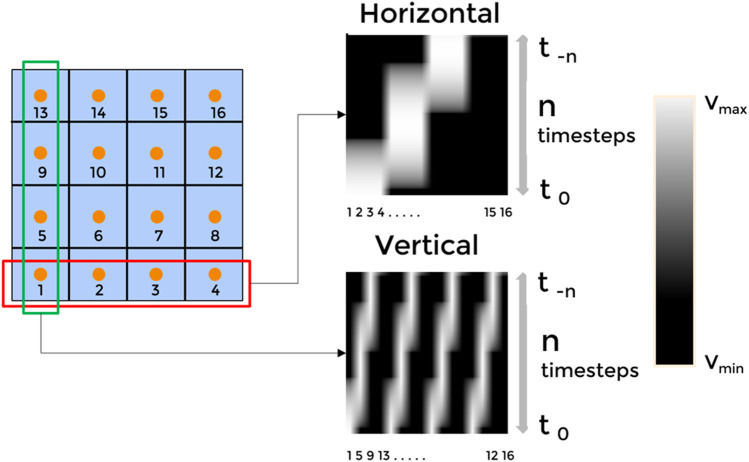
The tactile sensor comprises 16 taxels, numbered from 1 to 16. The data from these taxels can be flattened and vectorized either along rows or columns. Time series corresponding to each row or column are then stacked over time to form a spatio-temporal representation. The right panel illustrates both horizontal and vertical vectorization strategies, where each row or column's temporal dynamics are encoded as image-like patterns.

We hypothesize that the input image of the tactile data can be reduced to latent representations. Using VAE, the N × 16 input data can be expressed by n × 4 data array, where N is the total time steps (temporal component) of the input sequence, and n is the dimensions of the latent parameter. The 16 columns are separated into groups of four based on horizontal visualization or vertical visualization (Figure 5). Each of the N × 4 data is down-sampled to an n x 1 array using a separate VAE network. We concatenate the down-sampled arrays to get an encoding with the shape n × 4. The temporal nature of the data (as discussed in Section 2.4.1) ensures that the VAE extracts spatio-temporal features.

We chose a VAE for dimensionality reduction because it learns a smooth, structured latent space that helps generalize tactile features. While VAEs can sometimes produce slightly blurrier outputs than standard autoencoders, we found the trade-off acceptable for our task.

#### 2.4.3 Residual upsampler

A study by Cheng et al. (2019) introduced a method to effectively use an encoder-decoder network with residual skip connections to obtain SR images from input blurred images. The up-sampling block contains the Residual In Residual Block (RIRB), which combines the data from the previous layers and concatenates it with the processed data. The input to the first RIRB block is the latent features from the encoder network. Subsequent RIRB blocks are connected to each other in a sequential manner. RIRB contains several Residual Channel-wise Attention Blocks (RCAB) (Zhang et al., 2018), a convolution layer, and a skip connection. RCAB adopts channel-wise attention mechanism to adaptively distinguish the significance of the channels of the input data to the RCAB Block. It gives more significance to some of the extracted features among the input channels. In our case, given that we are dealing with latent features of the tactile data, not all the features are equally important. RCAB helps in giving significance to certain features, unlike commonly used residual blocks wherein the features are treated fairly. In our implementation, we use similar blocks but use them as our up-sampler block (by adding a transpose convolution layer) to selectively extract features and get SR output. Thus, the RIRB block in our case works both as a selective feature extractor and a upsampler to decode the latent features to super-resolution outputs.

For upsampling, we used RIRB, which are effective at preserving fine details in super-resolution. Though alternatives like sub-pixel convolution or UNet could also work, the proposed setup is chosen to achieve good balance between performance and efficiency for real-time use.

### 2.5 Synthetic dataset generation

As mentioned earlier, we converted the raw tactile data obtained from robotic palpation over textured surfaces into a 4 × 4 tactile images by linearly interpolating the voltage data, from the tactile sensor to grayscale. This 4 × 4 images are then passed through the SuperTac network to obtain 16 × 16 super-resolved tactile images. However, to assess the quality of obtained 16 × 16 tactile images, we needed an ideal representation of the textured surface in the form of 16 × 16 image within the same 13 mm × 13 mm of tactile sensing area. Therefore, we created synthetic dataset of 16 × 16 image for each timestep. Knowing the speed of the of the finger palpation and geometry of the texture enabled us in generating the ideal 16 × 16 tactile image, with each pixel denoting 1/16th of the 3 mm × 3 mm tactile sensor. For each time-step, the location of the finger on the textured plate is known and thus accordingly the location of the centre points of the 256 pixels (16 × 16) is calculated and the height of the texture at the any position is taken from the 3D design file of the texture. The height is then linearly-interpolated to grayscale. Also, to make the ground truth more realistic, we have added certain noises like mechanical vibration noise as minor sensor or object movements can create wavy distortions in the readings. Also, pressure can also be inconsistent at some points while palpating over textures. Hence, mechanical vibration noise will incorporate those factors in the ground truth dataset. Further, we added thermal drift, EMI noise, quantization artifacts, and sensor crosstalk noises. It is to be noted that the time interval between subsequent data is taken to be constant i.e., 1/300 s.

## 3 Training pipeline

**FIGURE 6:**
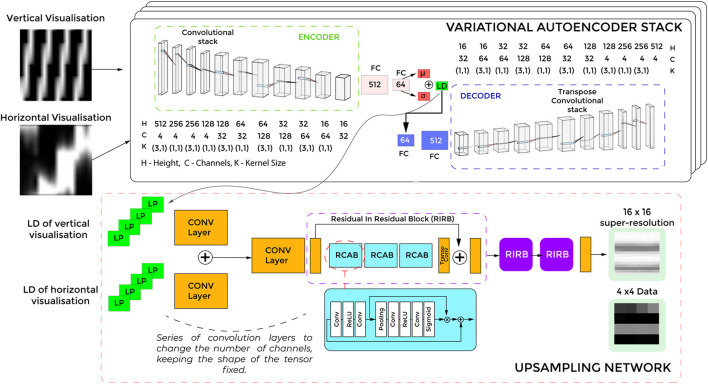
A schematic of the overall training pipeline of the Super-Tac network. As shown VAE stack is a stack of four identical VAE networks. The latent parameters (LP) of each from the four networks are the inputs to the upsampling network. The upsampling enlarges the latent features into super resolution data. FC -Fully connected layers.

The whole training pipeline, as shown in Figure 6 consists mainly of two parts, the VAE Stack and the Upsampling Network. We use an end-to-end approach to train the encodings and the super-resolution networks together. For the VAE stack, we use Kullback–Leibler divergence Loss (KLD Loss) and L2 loss for each VAE network of the stack. L2 Loss penalizes the model based on the Cartesian distance between the input and decoded images. KL divergence term in the loss function makes the distribution of the encoder output as close as possible to the standard multivariate normal distribution.

For the upsampling stack, we use L2 Loss and SSIM (Structural Similarity Index). L2 loss takes care of the Cartesian distance between the super-resolution output and the high-resolution ground truth data. The SSIM is a perceptual image measure, widely used for measuring performance of super-resolution networks. The overall loss of the network is a convex combination of losses from VAE stack and Upsampling network.

The Optimizer used in the process is the Adam optimizer. Our dataset consists of two main textures with a varying number of protrusions; bumps and ridges. The distances between the protrusions have been varied, as stated in Section 2.2.1. The dataset is split evenly between bumps and ridges. The tactile sensor has a sampling rate of 300 samples per second. We collected data over the 6 tactile stimuli (Figure 2) over two speeds of the robotic palpation; 5 mm/s and 10 mm/s. For each stimulus and speed pair, we performed eight sliding actions. This yielded us approximately 466000 numbers of 4 × 4 images.

## 4 Results

**FIGURE 7:**
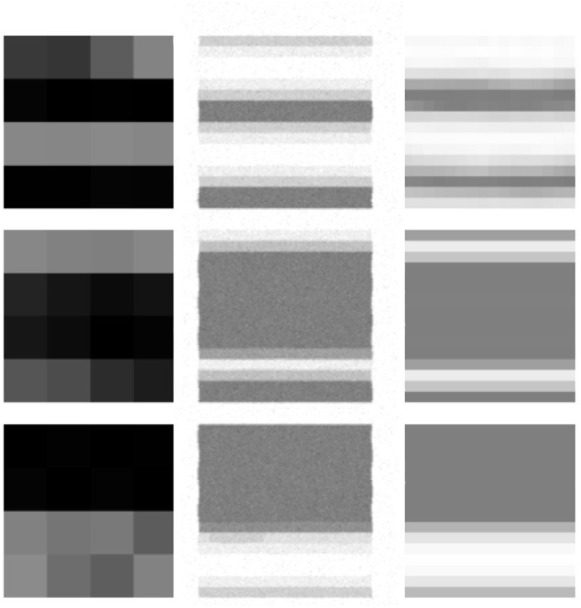
Results from the Super-Tac network. The left column denotes the 4 × 4 low resolution tactile data. The middle column indicates the simulated ground truth 16 × 16 high resolution image. The last column is the super- resolved output from the Super-Tac Network.

Figure 7 shows output of Super-Tac network. The input to the network is the tactile data of shape 4 × 4 (first column). The low-resolution data is upsampled to obtain super-resolution outputs denoted by the final column. Each of the output has a size of 16 × 16. The middle column is the simulated ground truth data. The texture plate used in this case consists of both bumps and ridges at varying distances from each-other.

In order to quantify our results, we use SSIM (Structural Similarity Index), PSNR (Peak Signal to Noise Ratio), and Phase Correlation as our metrics. These metrics are used to measure the performance of the SR tactile images to the simulated ideal high resolution tactile images. We also report the frames per second (FPS) of output SR images for the suitability of real-time usage of the proposed SuperTac algorithm.

### 4.1 SSIM and PSNR

Calculating the SSIM (Wang et al., 2004) and PSNR scores as a metric to determine the performance of super-resolution is a common practice (Wang et al., 2018; Ledig et al., 2017). Since our method involves visualizing the tactile data in an image-like format, we use the SSIM and PSNR metrics for our analysis.

$$PSNR=20\log_{10}\left(\mathrm{MAX}\right)-10\log_{10}\left(MSE\right)$$ (2)

$$SSIM\left(x,y\right)=\frac{\left(2\mu_{x}\mu_{y}+C_{1}\right)\left(2\sigma_{cy}+C_{2}\right)}{\left(\mu_{x}^{2}+\mu_{y}^{2}+1\right)\left(\sigma_{x}^{2}+\sigma_{y}^{2}+C_{2}\right)}$$ (3)

**FIGURE 8:**
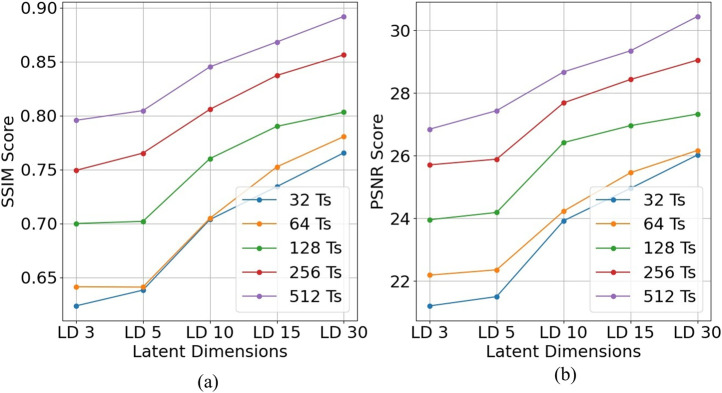
Reconstruction quality across latent dimensions and timesteps.**(a)** SSIM scores and **(b)** PSNR scores plotted against latent dimensions (LD) for different numbers of timesteps (T_s_). Each curve corresponds to a fixed timestep setting, as indicated in the legend.

In Equation 2, the MAX term represents the maximum possible value of each unit cell, while MSE denotes the mean squared error between the reconstructed and reference images. In Equation 3, the μ terms correspond to luminance (mean pixel intensity), and the σ terms capture contrast (standard deviation of pixel values). The constants C_1_ and C_2_ are introduced to ensure numerical stability, particularly when the luminance or contrast values approach zero. We plot (Figure 8) the average PSNR and SSIM by varying the latent dimensions. Each plotline in the graph is drawn, keeping the number of timesteps constant. We can see that both SSIM and PSNR score gradually ingresses. For the instant when the number of timesteps is 256, the SSIM increased from 0.749 to 0.856 Gradual increase in the SSIM score is due to larger, better, and richer encoding of the input image.

### 4.2 Frames per seconds (FPS)

The FPS is calculated by considering the data input (n x 16 image) as a frame at a particular time step. We calculate the FPS for all the cases by varying the number of latent dimensions (LD) and the number of timesteps to the network. We observe a gradual but minor increase in the inference speed with a decrease in the number of timesteps (Ts). Super-Tac processes all the data points in the range of 47.1–53.2 FPS (47.1 FPS for 512 Ts and 30 LD and 53.2 FPS for 30Ts and 3 LD), which suggests that the SR image of the input data can be generated in real-time given the FPS achieved are greater than 20 FPS. Therefore, we get the super-resolution output of the input image in real-time and can thus be used as a live feed when the network is employed on a prosthetic finger or a manipulator hand of an exploration robot. This also gives us almost 20 FPS overhead and thus allows further processing of the super-resolution output if needed and still makes the process real-time. FPS decreases, and PSNR values increase with the increase in latent dimensions, as shown in Table 1 and Figure 5, thus creating a tradeoff of speed v/s performance. This prompts us to consider the number of latent dimensions to be 10 for further analysis. This ensures that we get inference speeds almost close to the case of three latent dimensions but with performance closer to the case of 30 latent dimensions.

**TABLE 1:** Keeping the latent dimensions as 10, calculating the metrics SSIM, FPS and Phase Correlation (PC) by varying the timesteps.

Timesteps	SSIM	FPS	PC
512	0.84	48.89	88.76
256	0.80	50.79	94.32
128	0.76	51.24	93.32
64	0.70	51.83	86.27
32	0.70	52.76	85.38

### 4.3 Phase correlation

**FIGURE 9:**
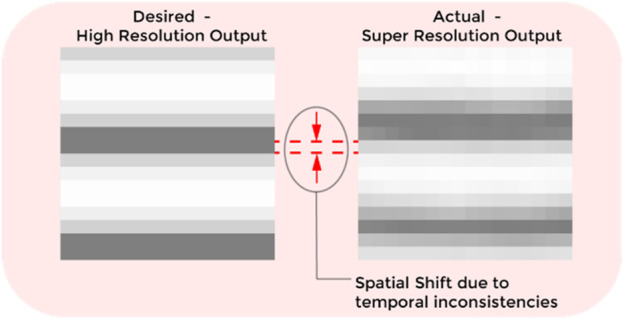
The spatial shift due to temporal inconsistencies in the simulated data and super-resolved output.

In some cases, despite achieving accurate inferencing, the PSNR and SSIM values remain low due to the construction of the SR tactile images and ground truth tactile images using binned data samples over fixed time periods. While generating the ground truth, we assume that the time interval between consecutive data points is constant. However, during palpation, there is an inherent margin of error in these intervals, causing them to vary (Figure 9). This variability introduces cumulative errors during data collection. Since SR images are generated from the collected data, which includes this time interval variability, whereas the ground truth relies on an idealized constant interval assumption, the PSNR and SSIM values are influenced by the duration of the binning period. This issue led us to adopt phase correlation as an alternative metric, as it is more robust to shifts between similar images.

We trained separate networks with varying timesteps: 512, 256, 128, 64, and 32, and analyzed the phase correlation (PC) values, which are presented in Table 1. We can see a bell-shaped relationship between timesteps and phase correlation in Table 1, with the highest performance observed at 256 timesteps (94.325 PC). This optimal performance strikes a balance between data resolution and smoothing, leading to the best accuracy in tactile data interpretation.

**FIGURE 10:**
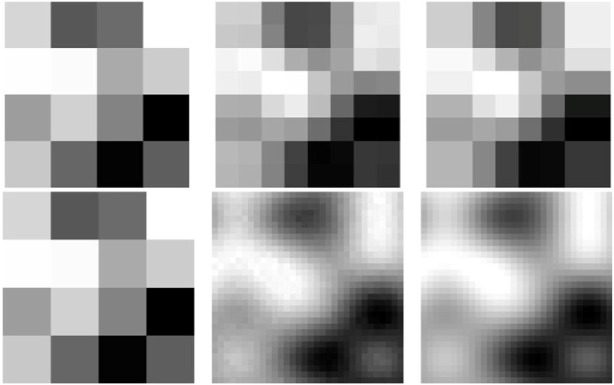
Result comparison for 2-fold (top row) and 8-fold (bottom row) super-resolution output. The first column represents input 4 × 4 low-resolution tactile data. The middle column indicates super-resolved output from the SuperTac network, and the last column represent the ground truth.

The above discussed results were obtained using a fourfold super-resolution (16 × 16) of the 4 × 4 input tactile image. To evaluate the effect of varying the super-resolution factor, we extended the analysis to include twofold (8 × 8) and eightfold (32 × 32) super-resolutions by modifying the SuperTac network accordingly. Figure 10 presents the qualitative results for these output resolutions. We noticed that 8 × 8 super-resolved output has less details i.e., it is limited in spatial resolution and 32 × 32 super-resolved image can introduce more artifacts, as the upscaling factor increases, small errors in estimation or interpolation get amplified, leading to artifacts like blurring, ringing or unrealistic patterns. Quantitative evaluation using SSIM and PSNR metrics, computed over 256-timesteps, is summarized in Table 2. The results indicate that both SSIM and PSNR peak at the 16 × 16 resolution, suggesting that a fourfold super-resolution offers the optimal balance between detail reconstruction and fidelity for the 4 × 4 input tactile data.

**TABLE 2:** Average PSNR and SSIM metric comparison for different resolution and constant timestep of 256.

Resolution	PSNR	SSIM
8 × 8	∼24.02	∼0.77
16 × 16	∼27.3	∼0.81
32 × 32	∼23.09	∼0.74

### 4.4 Texture classification

**FIGURE 11:**
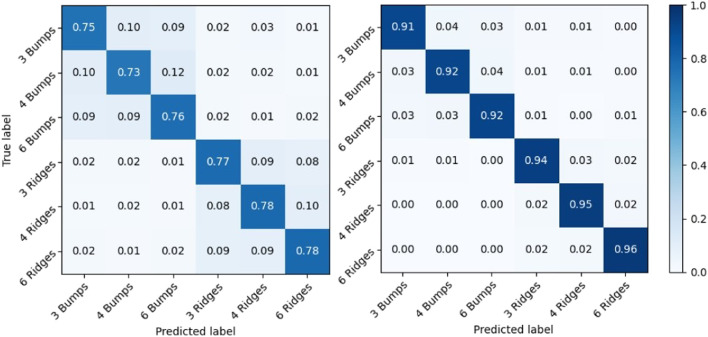
Confusion matrix for classification of artificial textures using 4 × 4 (Left panel) and 16 × 16 (Right panel) tactile data using CNN classifier.

In order to show the physical significance of super-resolved data from the SuperTac Network, we perform a comparative analysis of texture classification when the original 4 × 4 tactile data was used for classification compared to super-resolved 16 × 16 tactile data. We used a CNN based classifier network consisting of two convolution blocks and a softmax layer to classify different types of texture data. We varied the protrusions between bumps and ridges by 3,4, and 6 to create variation in textured plates. As shown in Figure 11, the classification network yields an accuracy of 76.3% when the 4 × 4 tactile data was used as input to the classifier, whereas using super-resolved 16 × 16 tactile data provided a classification accuracy of 93.1%. The 17% jump in texture classification accuracy when super-resolved tactile data was used for classification indicates the ability to capture fine details of textures by the SR tactile data obtained from the SuperTac network.

## 5 Discussion

The SuperTac framework presents an algorithmic approach to enhancing tactile resolution by leveraging spatiotemporal features of tactile data, enabling the reconstruction of high-resolution outputs from low-resolution inputs. Experimental results demonstrate improvements across multiple metrics, including SSIM, PSNR, and texture classification accuracy while maintaining real-time inference speeds. Unlike conventional approaches that rely on hardware-level enhancements, SuperTac achieves resolution enhancement through software-based processing. Although this introduces a marginal increase in energy consumption due to added computational load, it remains significantly lower than that of hardware-based solutions. This software-centric design offers a scalable and cost-effective alternative without modifying sensor hardware, making SuperTac particularly appealing for industrial applications in robotics, prosthetics, and haptic interfaces.

Compared to Wu et al. (2022), who adapted image-based CNNs and GANs for tactile super-resolution (TactileSRCNN and TactileSRGAN), SuperTac achieves comparable resolution enhancement while maintaining real-time processing speeds exceeding 50FPS, which is crucial for online deployment. While Wu et al.’s GAN-based methods provide high perceptual quality, their computational demands are significantly higher, making them less suitable for low-power embedded applications. Similarly, Park et al. (2021) presented EIT-NN, a neural reconstruction framework for EIT-based sensors. Unlike EIT-NN, which is tailored for a specific sensing modality, SuperTac is sensor-agnostic and does not require specialized hardware or calibration procedures, thus offering broader applicability across tactile platforms.

A key factor influencing the performance of the proposed algorithm is the interplay between the latent dimension and the choice of timestep, both of which govern the tradeoff between computational efficiency and output quality. The empirical results show that a latent dimension of 10 provides an effective balance offering near-optimal performance while maintaining high inference speeds suitable for real-time applications. Similarly, the relationship between timesteps and phase correlation (PC) follows a bell-shaped trend rather than a linear one. This can be explained by two competing factors. First, as the timestep decreases (e.g., from 256 to 32), the data resolution decreases, leading to less precise SR image construction, which increases positional error and decreases phase correlation. On the other hand, as the timestep increases (e.g., from 256 to 512), the binned data becomes overly smoothed, losing critical temporal variations needed to accurately capture tactile information, thus also reducing phase correlation. The optimal performance occurs at 256 timesteps, where the balance between data resolution and smoothing leads to the highest phase correlation value (94.325). This corresponds to an average positional error of 0.8 pixels (±0.65 mm) with respect to the desired high-resolution output. The results in Table 1 illustrate this relationship, emphasizing the importance of selecting an appropriate timestep to optimize the trade-off between resolution and error in tactile data processing.

In this study, we achieved 4 times super-resolution while maintaining the high degree of SSIM and PSNR, there remains potential to further improve performance through a broader and more diverse dataset or by incorporating a denser upsampling network. However, care must be taken to avoid overfitting, particularly as model capacity increases. Therefore, a well-balanced approach to model complexity and dataset diversity, ensuring generalization across various tactile scenarios is necessary.

The performance metrics used in this study are based on empirical evaluations using metrics such as SSIM, PSNR, and texture classification accuracy. To further strengthen the findings, incorporating statistical methods like hypothesis testing (e.g., t-tests or ANOVA) could be beneficial. Although not included in the current study, such methods represent a valuable direction for future work to enhance the statistical rigor and reproducibility of results.

The proposed SuperTac framework is designed to be sensor-agnostic and can, in principle, be applied to different types of tactile sensors. However, certain modifications may be necessary depending on the nature of the sensor data. For example, sensors with higher native resolution or different spatial layouts may require changes in the input encoding or network architecture. Similarly, sensors that capture multi-dimensional data (e.g., force vectors or shear) may benefit from a more complex feature extraction module. Despite these differences, the core idea of using dimensionality reduction to capture spatiotemporal features followed by learned upsampling remains applicable across sensor modalities.

## 6 Conclusion

In this study, we demonstrated the ability of the SuperTac framework to enhance tactile resolution through a dimensionality reduction network combined with residual upsamplers. The framework processes spatiotemporal information and generates high-resolution tactile images, as indicated by improved SSIM, PSNR, and FPS metrics. The ability to achieve super-resolution in real-time opens up significant potential for applications in robotics and prosthetics, where high-resolution tactile feedback is crucial for tasks like manipulation and exploration. Despite the promising results, real-world deployment may present additional challenges, including sensor noise, calibration issues, and environmental factors that could affect system performance. These challenges need to be addressed for robust operation in real-world scenarios. The SuperTac framework offers a scalable software-based solution for tactile super-resolution, distinguishing itself from hardware-based approaches used in commercial tactile sensors. This makes it a promising candidate for industry applications in robotics, prosthetics, and other areas requiring enhanced tactile perception. In future work, we plan to explore more diverse datasets and denser upsampling networks to further improve performance. Additionally, real-world validation and statistical analysis could provide deeper insights into the framework’s capabilities and potential for broader adoption.

## Data Availability

The raw data supporting the conclusions of this article will be made available by the authors, without undue reservation.
